# Disease Management of Early Childhood Caries: ECC Collaborative Project

**DOI:** 10.1155/2014/327801

**Published:** 2014-03-03

**Authors:** Man Wai Ng, Francisco Ramos-Gomez, Martin Lieberman, Jessica Y. Lee, Richard Scoville, Cindy Hannon, Peter Maramaldi

**Affiliations:** ^1^Boston Children's Hospital, Harvard School of Dental Medicine, Boston, MA 02115, USA; ^2^Section of Pediatric Dentistry, UCLA School of Dentistry, Los Angeles, CA 90095, USA; ^3^Neighborcare Health, 6200 13th Avenue South, Seattle, WA 98103, USA; ^4^Department of Pediatric Dentistry, University of North Carolina School of Dentistry, Chapel Hill, NC 27599, USA; ^5^Health Policy Management, Gillings School of Global Public Health, University of North Carolina, Chapel Hill, NC 27510, USA; ^6^DentaQuest Institute, 2400 Computer Drive, Westborough, MA 01580, USA; ^7^Simmons School of Social Work, 300 The Fenway, Boston, MA 02115, USA

## Abstract

Until recently, the standard of care for early childhood caries (ECC) has been primarily surgical and restorative treatment with little emphasis on preventing and managing the disease itself. It is now recognized that surgical treatment alone does not address the underlying etiology of the disease. Despite costly surgeries and reparative treatment, the onset and progression of caries are likely to continue. A successful rebalance of risk and protective factors may prevent, slow down, or even arrest dental caries and its progression. An 18-month risk-based chronic disease management (DM) approach to address ECC in preschool children was implemented as a quality improvement (QI) collaborative by seven teams of oral health care providers across the United States. In the aggregate, fewer DM children experienced new cavitation, pain, and referrals to the operating room (OR) for restorative treatment compared to baseline historical controls. The teams found that QI methods facilitated adoption of the DM approach and resulted in improved care to patients and better outcomes overall. Despite these successes, the wide scale adoption and spread of the DM approach may be limited unless health policy and payment reforms are enacted to compensate providers for implementing DM protocols in their practice.

## 1. Background

Early childhood caries (ECC) is a common childhood disease in the United States (US) and worldwide [1]. Until recently, the standard of care for ECC has been primarily surgical and restorative treatment with relatively little emphasis on the prevention and management of disease [2]. In the US, young children who are not cooperative are commonly sedated or treated under general anesthesia (GA). However, despite receiving costly treatment under GA, such as in the operating room (OR) [3–5], children all too often develop new and recurrent caries [6–10]. It is now accepted that surgical repair alone does not address the underlying etiology of the disease [11]. Unless the caries balance is altered, new and recurrent caries are likely to occur [12]. On the other hand, a successful rebalance of risk and protective factors may slow down or completely halt the disease process, resulting in caries arrest, if not also preventing the onset of new disease [13].

Contemporary approaches to caries prevention and management modeled after medical management of chronic conditions such as diabetes, obesity and asthma, have been described in the scientific literature and are herein known as chronic disease management (DM) [11, 13–15]. DM differs from the traditional approach of oral health care providers relying on a surgical treatment model in response to the disease, while telling the patients what to do. Instead, it assumes that patients have a central role in determining the care of their chronic condition [13]. A close collaboration between the healthcare provider and patient is required, ideally in a culturally and linguistically appropriate manner. In practice, healthcare providers coach patients and parents about the factors that lead to and protect against dental disease and assist them in selecting self-management goals to improve their own and their children's risk for disease. Treatment decisions are based on the latest evidence-based guidelines that are customized to patients' individual needs. Risk-based DM of ECC requires significant patient and family engagement and empowerment from the provider and care team in effective day-to-day behavior modifications (e.g., tooth brushing, topical fluorides, and dietary control) that address disease etiology [13]. Family-centered behavior plans lead to real behavior change and maintenance of oral health behaviors in the child's home. At the same time, the dental practice has a reciprocal role in tracking and managing the care of patients.

### 1.1. ECC Quality Improvement Learning Collaborative

**Phase 1**

In 2008, a risk-based DM approach to address preschool children with ECC was implemented and tested as a quality improvement (QI) demonstration project at Boston Children's Hospital in Boston, Massachusetts (BCH), and St. Joseph Health Services of Rhode Island in Providence, RI (SJH). The clinical protocol and project results have been previously published [13]. Thirty months of results found that children in the ECC group experienced lower rates of new cavitated lesions, pain, and referrals for restorative treatment under general anesthesia in the OR as compared to baseline historical controls (Table 1) [13]. At BCH, the ECC group experienced a 62% lower rate of new cavitation compared to the historical control group [13]. Structured interviews with Phase 1 parents revealed that most believed the DM approach to be helpful for their children; almost all parents appreciated given reasons as to why their children may have developed ECC. The collaborative approach allowed clinicians to engage parents or caregivers to better understand that they have a voice in the care their child receives [13].

**Phase 2**

Building upon the promising results of Phase 1, the project was expanded in 2011 to include five additional teams in the US.  Phase 2 further tested the feasibility and effectiveness of the DM approach to reduce ECC in more diverse settings. The five additional teams were in the following locations across the US: Holyoke Health Center (Holyoke, MA); Native American Health Center (San Francisco, CA); Nationwide Children's Hospital (Columbus, OH), Neighborcare Health (Seattle, WA); and University Pediatric Dentistry (Buffalo, NY). The purpose of this report is to describe the Phase 2 project and experiences, present the results after 18 months, and discuss the implications of what was learned.

### 1.2. Structure of Phase 2

Phase 2 was implemented as an 18-month QI Learning Collaborative. Using established QI methods, a nationwide collection of staff, experts, and faculty provided training and technical assistance to the seven participating teams, which included the two teams that were part of Phase 1. Teams were required to attend three on-site “learning sessions” where each of the seven teams received didactic education and training on QI concepts and activities. The learning session curriculum focused on the use of logic models, measurement plans, Plan-Do-Study-Act (PDSA) cycles, DM of ECC such as caries risk assessment (CRA), self-management goals (SMGs), effective patient-provider communication, and fluorides and other remineralizing modalities. Teams learned from each other by sharing their experiences, successes and struggles. The learning sessions provided invaluable opportunities for synergy as teams exchanged approaches to DM during consultations with experts, faculty, and staff, who provided coaching and support.

QI has been defined as the combined and unceasing efforts of everyone to make changes that will lead to better patient outcomes (health), system performance (care), and professional development (learning) [16]. QI is intended to support the redesign of care processes based on a system of learning, incremental change, and the incorporation of empirically supported best practices from evaluating performance and outcome measures. Unlike a protracted randomized trial, QI uses systematic, data-guided activities designed to bring about immediate improvements in health care delivery in particular settings [16] and can be considered as the scientific method used for action-oriented learning.

**Figure 1:**
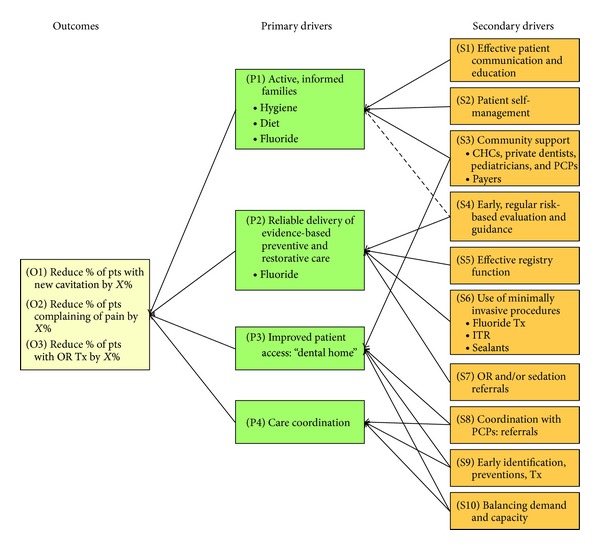
ECC Phase 2 Driver Diagram.

The Model for Improvement [17] developed by Associates in Process Improvement was used as the essential framework to guide changes made by each team's care delivery system in order to use a DM approach to address ECC. The Collaborative developed a driver diagram outlining three main outcomes of interests: (1) new cavitation; (2) pain related to untreated caries; and (3) referral to the OR, along with primary and secondary drivers affecting those outcomes (Figure 1).

PDSA [17] cycles are small-scale tests of change in real work settings—by planning a test, trying it, observing the results, and acting on what is learned. PDSA cycles promote creativity, offer quick results, and empirically support approaches to DM that are specific to the clinical teams. For example, PDSAs served as learning opportunities for Phase 2 teams to use evidence to determine how to perform activities such as CRA, SMGs, and patient scheduling to support the additional DM visits required.

### 1.3. ECC DM Protocol

**Figure 2:**
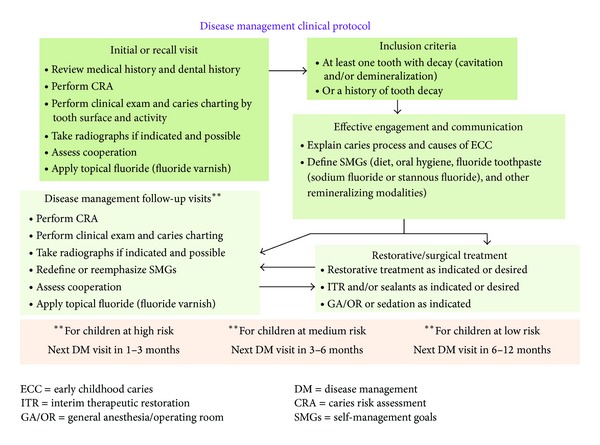
ECC Phase 2 disease management clinical protocol.

The Phase 2 clinical DM protocol (shown in Figure 2) were modeled after Phase 1. The ECC DM approach assumes that caries risk can change over time.

**Figure 3:**
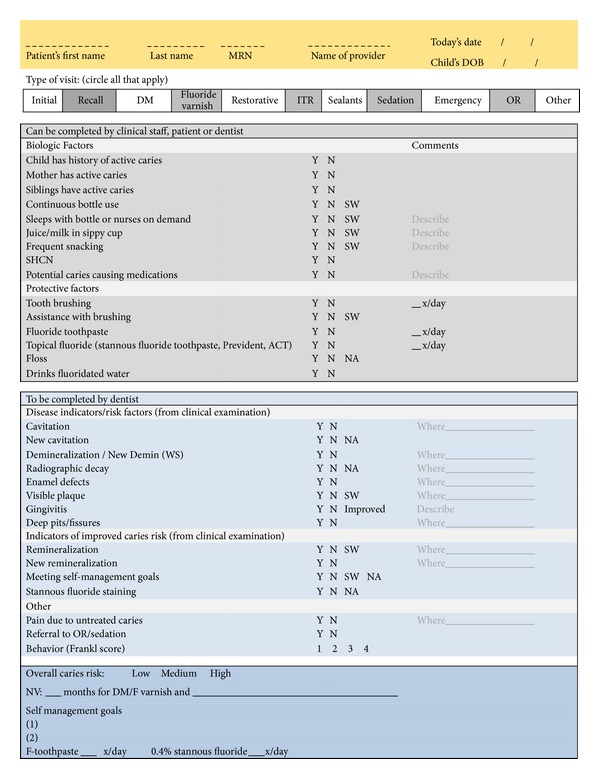
Sample ECC Phase 2 caries risk assessment form.

CRA and SMGs in combination are the cornerstones of DM approach. CRA allows for a customized prevention and maintenance plan to be developed that is appropriate for the child and the family. CRA involves asking parents a few questions to assess each child's risk for caries at the initial visit and every visit thereafter.  Figure 3 shows an example of a CRA form used in Phase 2. This form was adapted from the American Academy of Pediatric Dentistry (AAPD CRA) form and the pediatric Caries Management by Risk Assessment (CAMBRA) form. Teams were able to customize forms for use with their specific patient populations and organizational context provided that they included at least the basic questions seen in Figure 3. 

#### 1.3.1. DM Clinical Protocol

Children who had at least one tooth with clinical manifestation of caries—tooth decay (including demineralization)—or who had a history of carious lesions was considered an ECC patient. At the initial and recall visits, the medical and dental history were reviewed. A clinical examination and charting were performed to allow for the tracking of caries presence and activity by tooth and surface, since decay may progress and become inactive at different sites of the dentition at the same time.

**Figure 4:**
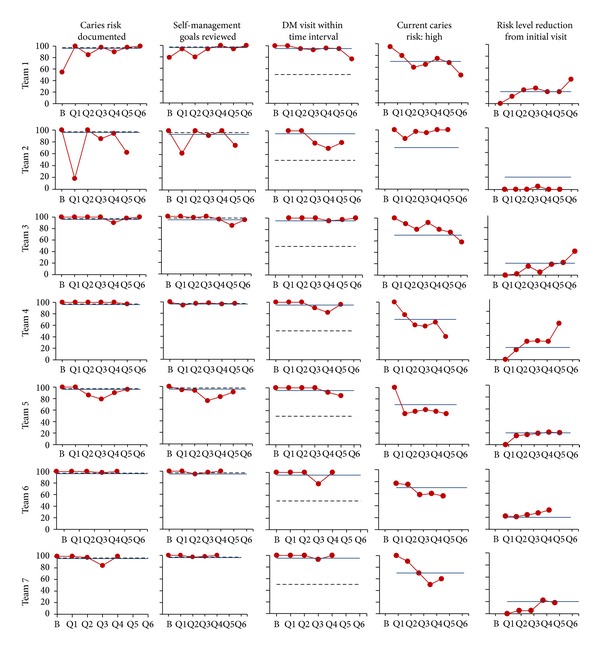
Run charts showing some of the trend data for the ECC Phase 2 teams.

Parents of ECC patients were engaged and coached about the factors that lead to caries and tooth decay by dentists, hygienist, dental assistants, and/or support staff. Parents learned about the caries process as they were informed of the ways that tooth decay can be prevented and stopped. In addition, parents learned that without a change in diet and home care, new cavities and broken fillings will likely result. Providers and care team members worked with parents to select SMGs to improve their child's disease risk.  Figure 4 presents an example of a SMGs handout used in Phase 2. Such goals include basic caries control strategies such as more frequent tooth brushing, using topical fluorides at home, and modifying one's diet to include fewer and less frequent intake of sugary products.

The frequency of return DM visits for patients and parents—in-office and at the clinic site—was based on their caries risk. Whenever possible, the DM activities were coordinated with restorative treatment.  Table 2 shows the DM protocol with return visit intervals based on the most recent caries risk status in conjunction with restorative care as needed and as desired by the parent and dental provider.

The in-office DM protocol was based on the assumption that children who initially presented as high caries risk may improve their risk over time. Children who were assessed to be high caries risk were advised to return in 1–3 months for a DM visit. Medium or moderate risk children returned in 3–6 months, while low risk children returned in 6–12 months. In some cases, accurate clinical assessment was hampered by the presence of heavy plaque and/or a lack of patient cooperation. As a result, a one-month follow-up visit for a child assessed to be high caries risk allowed for a more accurate assessment of demineralized, cavitated, and remineralized tooth surfaces.

During each recall or subsequent DM visit, a CRA was performed. Providers asked parents to report on their experiences with the SMGs in order to assess the level of compliance and the utility of the agreed-upon SMGs. A clinical examination was also performed, reassessing for the presence of new demineralization and cavitation along with caries remineralization. All findings were recorded. Intraoral radiographs were taken if indicated and possible, and fluoride varnish was applied.

#### 1.3.2. Restorative Treatment

Parents were given the full range of options for restorative treatment, which included pharmacologic management (i.e., use of nitrous oxide, sedation, or GA/OR) as needed by the patient and as desired by the parent. Restorative options included conventional treatment and minimally invasive restorative treatment (i.e., interim therapeutic restorations (ITR)). If the destruction of the tooth structure by the caries process was minimal, caries arrest was possible with remineralization of the tooth structure. The restorative treatment was then deferred in patients if the caries process was stabilized, especially in a child unable to cooperate for restorative treatment. However, close follow-up and preventive care based on caries risk were essential to safeguard from relapse. If the decay had progressed into dentin and caries arrest was not achieved, ITR was offered as an alternative treatment with early cavitated lesions. Parents were informed that this was caries control rather than permanent restoration. A secondary gain from more frequent visits for preventive care was usually a reduction in a child's fears and a gain in trust between the dental provider and the child over time, allowing for restorative treatment to be completed with greater ease at a later time.

### 1.4. Practice Redesign to Support Disease Prevention and Management of Caries

In order for teams and their sites to support risk-based disease prevention and management of ECC, a redesign of their care delivery systems was needed. Dentists, staff, patients, and families who were accustomed to conventional surgical and restorative care were educated about and guided to accept a contemporary approach that emphasizes risk assessment, individualized disease prevention, and management and maintenance of health. For example, scheduling systems, typically set up to accommodate recall preventive visits every six months as allowed by insurance, had to be adjusted to allow for more frequent preventive return visits for high caries risk patients.

Before and during the implementation of the project, senior leaders and clinical champions of each team provided training to their dental providers and staff about the DM protocol. They shared what they learned from the Learning Sessions and monthly calls on DM and QI methods with those who were involved in the day-to-day work of implementing the ECC DM protocol. Teams were expected to hold regular meetings to address questions about the protocol and care management of patients, review project progress, plan PDSAs, and institute change. Most teams chose to begin the protocol initially with a few providers, followed by spreading to additional providers over time.

## 2. Methods

### 2.1. Measurement Plan and Data Collection

In Phase 2, teams collected process and outcome measurement data for the purpose of evaluating improvement trends in care processes and patient outcomes over time. Each month, teams randomly selected 20 patient records (charts) of their ECC patients to record the results of some measures. Meanwhile, on a quarterly basis, they selected 30 charts of ECC patients to record results of other measures. The teams submitted these data to the Collaborative each month without using patient identifiers. The deidentified data were collected and managed using REDCap [18] (Research Electronic Data Capture) electronic data capture tools hosted at BCH. REDCap is a secure, web-based application designed to support data capture for research studies providing (1) an intuitive interface for validated data entry; (2) audit trails for tracking data manipulation and export procedures; (3) automated export procedures for seamless data downloads to common statistical packages; and (4) procedures for importing data from external sources. BCH staff retrieved and processed the data, screened for errors, and managed the deidentified data.

Each month, run charts were produced by BCH and sent to the Collaborative staff. In turn, the Collaborative sent run charts to each team for use in monitoring progress toward improvement. During monthly Collaborative calls, trends in the run charts were reviewed. In addition, any questions and concerns from the teams were addressed by the Collaborative staff, faculty, and Improvement Advisor.

Although this Collaborative was designed as a QI initiative that aimed to identify positive trends in process and outcome measures which would signify improvements in care and outcomes, a trends analysis would not necessarily infer causality. Therefore, there was a need to compare the project outcome data to baseline data derived from historical controls (i.e., patients treated by the teams prior to the start of Phase 2). In the last several months of the Collaborative, after obtaining IRB approval, teams collected data, on the three outcomes of interest (percentage of patients with new cavitation, pain, and unplanned referrals to the OR) by randomly selecting 50 charts of their ECC patients and 50 charts of baseline historical control patients. At each site, a computer generated randomized scheme identified the potential ECC and control patients.

Qualifying ECC patients were those who had been (a) in Phase 2 for a minimum of 6 months and (b) had at least one formally scheduled preventive visit (recall visit) whereby caries charting was performed and documented. All sites reviewed their Phase 2 records (i.e. Excel spreadsheets or billing generated report) and randomly selected 50 ECC patients (one site had only 46 patients).

Qualifying historical control patients were children who were younger than 60 months of age and had (1) a history of decay; (2) at least one recall visit six months after the initial visit; and (3) a last visit that was at least six months prior to the start of the Phase 2.

For each team, a computer generated randomized scheme identified potential patients based on age and whose billing records were reviewed to determine whether they had a recall visit at least 6 months prior to the start of Phase 2. These patient records were selected for further review to determine those patients who met the qualifying criteria.

The following information was documented for both ECC and historical control patients by visit date: (1) type of visit (preventive, restorative, sedation, OR, missed, or canceled), (2) new cavitation identified, (3) pain identified, and (4) referral to OR. The first visit was determined for each patient as that which decay was initially charted or documented in the patient's clinical notes. Pain and OR referral at first visit, including pain unrelated to untreated decay at any visit were excluded. From this information, the percentage of patients with new cavitation, pain identified, and referral to OR were determined.

The ECC and historical control data were collected by most teams onto paper collection forms. The forms were sent to BCH for data entry into a separate REDCap database. One team entered their data directly into the REDCap database.

## 3. Results

Figure 4 shows some of the trend data for the seven Phase 2 teams. Over time, the teams demonstrated a highly consistent level of performance with their providers performing CRAs and SMGs. Most teams saw a reduction of ECC children deemed as high caries risk and an increase in ECC children with improved caries risk from the first visit.

Table 3 shows a comparison of the rates of new cavitation, pain, and referral to OR between ECC patients and the baseline patients for Phase 2. These results reflect a random sample of 316 historical control children and 344 ECC children drawn from a total Phase 2 population of 3,030. In the aggregate, children in the ECC DM group experienced lower rates of new cavitated lesions, pain, and referrals for restorative treatment under general anesthesia in the operating room (OR) as compared to baseline historical controls, although there was variability from site to site.

## 4. Discussion

In Phase 2 of the ECC Learning Collaborative, by using QI methods to change their systems of care, teams were able to efficiently implement the DM protocol into their clinical practice. In the aggregate for Phase 2, fewer ECC children experienced new cavitation, pain, and referrals to the OR compared to baseline historical controls.

There was discrepancy from team to team in the degree of improvement in the process measures and outcomes achieved. The variation in outcomes among teams, as expected, may be attributed to differences in each team's use of the DM protocol or to distinct cultural and socioeconomic differences in patients and families among the sites. Similarly, Phase 1 also found imbalance in terms of outcomes achieved at the two sites. The Phase 1 team that demonstrated a relatively less dramatic improvement in new cavitation rates had predominantly English speaking providers serving predominantly Latino populations who spoke Spanish as their native language. Unfortunately, demographic data were not collected in Phase 2.

In terms of limitations, 50 randomly selected charts for each of the ECC and historical control groups at each site may be insufficiently representative of the groups for each team. At the same time, an 18-month follow-up period may be an inadequate length of time to evaluate the clinical outcomes of some children who were “enrolled” as ECC patients over time. In addition, although dental providers at all sites received training on the DM protocol to chart decay (by using a modified ICDAS system [19]), they did not receive calibration to chart new cavitated or precavitated lesions. However, by protocol, ECC patients were seen more frequently for DM visits, during which time they were examined for new cavitation and thus may have received increased opportunities for new cavitated lesions to be identified.

During their participation in Phase 2, teams shared their experiences including their knowledge and skills gained, lessons learned, and tools developed with other Collaborative participants. Examples of new skills include training of support staff and employing them to assist with CRA and patient education, collaborating with pediatric medical providers to enhance the referral of young children for early preventive dental visits, scheduling differently to accommodate the more frequent return needs of the ECC children for DM visits, and managing no-show appointments by using a registry to track patient visits. Two teams were located in community health centers that initially saw a limited number of young children. These teams developed PDSAs to specifically focus on increasing referrals from pediatricians within their centers, and they were successful in their efforts. One site had a baby's clinic already in place prior to joining Phase 2 and a hygienist to see children specifically younger than age three years for infant oral health visits; this hygienist was already using a CRA tool. This site incorporated the DM protocol, SMGs, and more frequent visits first into their baby's clinic and later spread the DM protocol to other providers in their main dental clinic over time. Tools developed, enhanced, and willingly shared among the Collaborative teams included forms for conducting and documenting carious lesions, CRA, SMGs, and tracking of the ECC patients.

At the conclusion of Phase 2, team leaders convened for a final summary conference. All teams agreed that the DM approach to address ECC was a logical change in practice, albeit not easy to implement. Each team faced challenges that were especially formidable early on. Challenges included having to accommodate the additional DM visits and the time required for each visit. In some cases, 15 minutes could be added to a restorative visit. Initially, most providers and teams struggled with having to fit in the DM visits, especially if their schedules were booked out in advance. Since most dental insurance plans do not cover more than two diagnostic/preventive visits each year, the additional DM visits and lost time for reimbursable restorative care posed as additional obstacles. Systematic testing of new approaches (via PDSAs) helped to overcome barriers. Changes that worked were implemented across the practice and continually improved upon through further testing. Providers received training and coaching to be able to perform CRA, explain the causes of the caries process, and work with the caregivers to select SMGs more effectively and with greater efficiency. Providers and families began to accept a paradigm shift that addressed disease etiology in lieu of relying solely on a surgical model of treatment.

The use of QI methods was useful in facilitating the adoption of DM to address ECC, first by getting buy-in from the early adopters at each site, followed by later adopters. Some teams had spread the protocol to more providers within their primary site, while others had spread it to all their providers. One team successfully spread the protocol beyond their primary site to other dental sites that were a part of their community center network. Most sites embedded their DM protocols into their systems of care such that dismantling those systems would require effort. Most team affirmed a desire to continue using the DM approach. When the team leaders were asked “What impact did the Collaborative have on you?” responses included “It made me a better provider, a better teacher;” and “I no longer view children 0–5 the same way (I do not pick up the hand-piece first).”

## 5. Conclusion

We demonstrated the feasibility of an innovative approach to address ECC utilizing DM protocols that can be successfully implemented into dental practice using QI methods in a learning collaborative model. Although not easy to implement, after 18 months, all teams reported that the DM approach resulted in overall improved care delivery and patient outcomes (new cavitation, pain, and referrals to the OR). Teams recognized that while a DM model can be implemented into practice, policy and payment reforms are needed to facilitate a wider-scaled adoption of the DM protocols. Elements to be addressed include compensation for providers' time and efforts to perform CRA, SMGs, patient education and engagement, and the DM visits. Future demonstration should quantify opportunities for cost savings to be realized by avoiding more costly restorative treatment. Future policy changes are necessary to support a paradigm shift from surgical treatment of caries toward an individualized risk-based disease prevention and management model as the new standard of care. At the same time, the use of QI methods may help accelerate the adoption and spread of DM protocols into any dental practice.

## Figures and Tables

**Table 1:** ECC Phase 1: comparison of rates of new cavitation, pain, and referral to OR between ECC patients and historical controls.

Outcomes	BCH			SHS		
	ECC (*N* = 403) %	Historical control (*N* = 129) %	Improvement %	ECC (*N* = 234) %	Historical control (*N* = 80) %	Improvement %
New cavitation	26.1	75.2	65.3	41.0	71.3	57.5
Pain	13.4	21.7	38.2	7.3	31.3	23.3
Referral to OR	10.9	20.9	47.8	14.9	25.0	67.8

**Table 2:** ECC Phase 2: disease management protocol.

Existing risk category	New clinical findings	Fluoride varnish interval	Self-management goals	Restorative treatment	DM return interval	Other
Low	(i) No disease indicators of caries (ii) Completely remineralized (arrested) carious lesions	6–12 months	(i) Twice daily brushing with F toothpaste^†^ (ii) Stannous fluoride^‡^ on cavitated lesions		6–12 months	

Medium	(i) No disease indicators* but has risk factors** and/or inadequate protective factors*** (ii) Disease indicators present with some remineralization	3–6 months	(i) Twice or more daily brushing with F toothpaste^†^ (ii) Stannous fluoride^‡^ on cavitated lesions (iii) Dietary changes	(i) Sealants (ii) ITR (iii) Conventional Restorative	3–6 months	(i) Xylitol gum or candies or wipes (ii) Calcium phosphate paste

High	(i) Active caries (disease indicators present) (ii) No remineralization occurring (iii) Heavy plaque	1–3 months	(i) Twice or more daily brushing with F toothpaste^†^ (ii) Stannous fluoride^‡^ on cavitated lesions (iii) Dietary changes	(i) ITR (ii) Sealants (iii) Conventional restorative	1–3 months	(i) Xylitol gum or candies (ii) Calcium phosphate paste

ECC: early childhood caries; DM: disease management; ITR: interim therapeutic restoration.

*Examples of disease indicators include demineralization, cavitated lesions, existing restorations, enamel defects, deep pits, and fissures.

**Examples of risk factors include patient/maternal/family history of decay, plaque on teeth, and frequent snacks of sugars/cooked starch/sugared beverages.

***Examples of protective factors include fluoride exposure (topical and/or systemic) and xylitol.

^†^Brush with a smear of 1000 ppm F toothpaste.

^‡^Apply a smear of 1000 ppm stannous fluoride to the cavitated lesions.

**Table 3:** ECC Phase 2: comparison of rates of new cavitation, pain, and referral to OR between ECC patients and historical controls.

Outcomes	ECC (*N* = 344) %	Historical control (*N* = 316) %	Percentage improvement %	Improvement range %
New cavitation	33	46	28	14–71
Pain	8	11	27	80–100
Referral to OR	14	22	36	0–81
